# Cardiopulmonary exercise testing before and after intravenous iron in preoperative patients: a prospective clinical study

**DOI:** 10.1186/s13741-023-00319-x

**Published:** 2023-07-03

**Authors:** James O. M. Plumb, James M. Otto, Shriya B. Kumar, Sitara Bali, Mai Wakatsuki, Walter F. J. Schmidt, Hugh E. Montgomery, Michael P. W. Grocott, Denny Z. Levett

**Affiliations:** 1 Perioperative and Critical Care Theme, NIHR Southampton Biomedical Research Centre, University Hospital Southampton NHS Foundation Trust/University of Southampton, Southampton, UK; 2 Centre for Human Integrative Physiology, Faculty of Medicine, University of Southampton, Southampton, UK; 3 Anaesthesia and Critical Care Research Unit, University Hospital Southampton NHSFT, Southampton, UK; 4 Shackleton Department of Anaesthesia, University Hospital Southampton NHSFT, Southampton, UK; 5 Department of, Sports Medicine/Sports Physiology, University of Bayreuth, Bayreuth, Germany; 6 Centre for Human Health and Performance/Institute of Sport, Exercise and Health, University College London, London, UK; 7 NIHR University College London Hospitals Biomedical Research Centre, London, UK; 8 Department of Anesthesiology, Duke University School of Medicine, Durham, NC USA

**Keywords:** Anemia, Intravenous iron, Cardiopulmonary exercise testing, CPET, Total hemoglobin mass, tHb-mass, Surgery

## Abstract

**Background:**

Anemia is associated with impaired physical performance and adverse perioperative outcomes. Iron-deficiency anemia is increasingly treated with intravenous iron before elective surgery. We explored the relationship between exercise capacity, anemia, and total hemoglobin mass (tHb-mass) and the response to intravenous iron in anemic patients prior to surgery.

**Methods:**

A prospective clinical study was undertaken in patients having routine cardiopulmonary exercise testing (CPET) with a hemoglobin concentration ([Hb]) < 130 g^.^l^−1^ and iron deficiency/depletion. Patients underwent CPET and tHb-mass measurements before and a minimum of 14 days after receiving intravenous (i.v.) Ferric derisomaltose (Monofer®) at the baseline visit. Comparative analysis of hematological and CPET variables was performed pre and post-iron treatment.

**Results:**

Twenty-six subjects were recruited, of whom 6 withdrew prior to study completion. The remaining 20 (9 [45%] male; mean ± SD age 68 ± 10 years) were assessed 25 ± 7 days between baseline and the final visit. Following i.v. iron, increases were seen in [Hb] (mean ± SD) from 109 ± 14 to 116 ± 12 g l^−1^ (mean rise 6.4% or 7.3 g l^−1^, *p* =  < 0.0001, 95% CI 4.5–10.1); tHb-mass from 497 ± 134 to 546 ± 139 g (mean rise 9.3% or 49 g, *p* =  < 0.0001, 95% CI 29.4–69.2). Oxygen consumption at anerobic threshold ($\dot{\text{V}}$ O_2 AT_) did not change (9.1 ± 1.7 to 9.8 ± 2.5 ml kg^−1^ min^−1^, *p* = 0.09, 95% CI − 0.13 − 1.3). Peak oxygen consumption ($\dot{\text{V}}$ O_2 peak_) increased from 15.2 ± 4.1 to 16 ± 4.4 ml^.^kg^.−1^ min^−1^, *p* = 0.02, 95% CI 0.2–1.8) and peak work rate increased from 93 [67–112] watts to 96 [68–122] watts (*p* = 0.02, 95% CI 1.3–10.8).

**Conclusion:**

Preoperative administration of intravenous iron to iron-deficient/deplete anemic patients is associated with increases in [Hb], tHb-mass, peak oxygen consumption, and peak work rate. Further appropriately powered prospective studies are required to ascertain whether improvements in tHb-mass and performance in turn lead to reductions in perioperative morbidity.

**Trial registration:**

ClinicalTrials.gov identifier: NCT 033 46213.

**Supplementary Information:**

The online version contains supplementary material available at 10.1186/s13741-023-00319-x.

## Introduction

Anemia, defined by the World Health Organization (WHO) as a haemoglobin concentration of ([Hb]) < 130 g^.^l^−1^ in men and < 120 g^.^l^−1^ in women, is identified in around 30% of elective surgical patients and is associated with adverse perioperative outcomes (Baron et al. 2014). However, it is unclear whether this association is directly causal (Fowler et al. 2015); anaemia may be associated with more severe or advanced disease, for instance, or with increased requirements for blood transfusion (which may independently impact outcome) (Baron et al. 2014; Musallam et al. 2011).

One mechanism by which anaemia may directly impact surgical outcome is through its effects on aerobic exercise capacity. Poorer preoperative exercise capacity is associated with a greater risk of complications and a higher mortality rate (West et al. 2016; Snowden et al. 2010). Maximal oxygen uptake ($\dot{\text{V}}$ O_2 max_) is dependent on adequate oxygen delivery to respiring tissues—a function of blood oxygen (O_2_) content (haemoglobin content and its saturation with oxygen) and blood flow (cardiac output and regional distribution). Whilst debated, cardiac output is believed to primarily limit maximal exertional oxygen consumption in otherwise healthy subjects (Wagner 2000). However, in the presence of severe anaemia, oxygen content may impact aerobic exercise capacity. The O_2_ carrying capacity of arterial blood is largely influenced by the haemoglobin content (or total haemoglobin mass, tHb-mass). [Hb] is determined by the plasma volume (PV) in which the tHb-mass is carried. As such, it shows a greater day-to-day fluctuation than does tHb-mass and consequently is a less precise measure of O_2_ carrying capacity (Otto et al. 2017b).

Cardiopulmonary exercise testing (CPET) represents the gold standard method by which $\dot{\text{V}}$ O_2 max_ is measured. Cross-sectional cohort studies have reported lower exercise capacity (peak oxygen uptake $,\dot{\text{V}}$ O_2 peak_) and anaerobic threshold ($\dot{\text{V}}$ O_2AT_) in anaemic elective surgical patients (Bartoszko et al. 2019). An increase in [Hb] should increase oxygen delivery to the tissues and might therefore affect the transition of aerobic to anaerobic glycolysis. However, this is not clear cut, with several studies conducted in athletes (Schmidt and Prommer 2010) and diabetic patients (Koponen et al. 2013) suggesting little correlation of [Hb] with oxygen consumption ($\dot{\text{V}}$ O_2_) at peak and at anaerobic threshold (Minto and Struthers 2017). One of the reasons that tHb-mass has a better correlation is due to the dual role it plays in this; on the one hand, it determines [Hb] in concert with the total blood volume but it also raises blood volume (thus potentially increasing cardiac preload and thus stroke volume) via erythrocyte volume and this double effect explains the superior correlation described by *Schmidt* and *Otto* (Minto and Struthers 2017; Otto et al. 2017b; Schmidt and Prommer 2010).

In elite athletes, all other elements of oxygen delivery are optimised and thus increasing blood volume and haemoglobin content have both improved $\dot{\text{V}}$ O_2 peak_ $, \dot{\text{V}}$ O_2AT_ and physical performance (Plumb et al. 2016b). This may not hold true in patients, amongst whom other factors may be limiting. Nonetheless, there are some (albeit limited) data to support a similar favourable impact on exercise capacity after blood transfusion in adult patients with stable haematological conditions (Wright et al. 2014) and in children with severe beta-thalassemia (Marinov et al. 2008). Exercise capacity also improves in patients with chronic heart failure and iron deficiency in response to intravenous (i.v.) iron (Van Veldhuisen et al. 2017). However, the effect of i.v. iron on CPET variables in anaemic preoperative patients remains unexplored. This is of importance given that impairments in CPET-derived physical fitness are associated with an increased risk of adverse postoperative outcome (Moran et al. 2016) and thus optimising functional status preoperatively may reduce these risks. It is unclear why and/or if the anaerobic threshold is affected by changes in oxygen content. This may be physiologically plausible via increases in exercise via other mechanisms or changes in cytochromes involved in electron transport.

The hypothesis that the association of anaemia with impaired perioperative outcomes may be in part mediated through impacts on physical fitness thus merits investigation. Therefore, we aimed to explore the relationship between exercise capacity, anaemia and haemoglobin content in an elective surgical cohort. The primary objectives were to obtain data relating to whether augmenting tHb-mass and [Hb] through intravenous iron therapy may improve oxygen consumption at anaerobic threshold ($\dot{\text{V}}$ O_2AT_) and peak oxygen consumption ($\dot{\text{V}}$ O_2 peak_) as quantified by CPET.

## Methods

The study took place at University Hospital Southampton (UHS) NHS Foundation Trust between February and October 2018. Ethical approval was granted by the London—Surrey Research Ethics Committee and NHS Health Research Authority (REC reference 17/LO/2061). Local permissions were received from the University of Southampton (ERGO ID: 31,688), University Hospital Southampton NHS Foundation Trust (R&D CRI 0357) and Southampton Centre for Biomedical Research Clinical Research Facility. The study was performed in accordance with the ethical standard set by the Declaration of Helsinki. Written informed consent was obtained from all participants. It was registered on ClinicalTrials.gov PRS with the unique Protocol ID—NCT 033 46,213.

Adults > 18 years (and over 50 kg) who were having preoperative CPET as part of routine clinical care were recruited. These patients were being worked up for possible major surgery and entered the perioperative pathway early via the anaemia service at UHS allowing recruitment in advance of surgical listing. At the time of the study, this was standard practice for the major UK teaching hospital at which the study took place. The authors are cognisant that this standard of care is not universal.

Additional inclusion criteria were anaemia with a [Hb] < 130 g^.^l^−1^ *and* iron deficiency (being iron restricted/deplete) *or* functional iron deficiency (see Additional file 1: Appendix 3 in Supplementary Material for exactly how this was classified). The use of < 130 g^.^l^−1^ was based on the Association of Anaesthetists of Great Britain and Northern Ireland (AAGBI) recommendations and our own local perioperative pathway, accepting that this differed from the WHO definition for female patients described in the introduction (Muñoz et al. 2017). It is noted that ‘functional iron deficiency’ does not have a universally agreed definition and that the guidelines used at the time of this study did not state an upper limit for ferritin; however, an upper limit off 600 ng.ml^−1^ is used in the institution where the study took place, and in this study, the highest level of ferritin recorded was 59 ng.ml^−1^ (Plumb et al. 2016a).

**Fig. 1:**
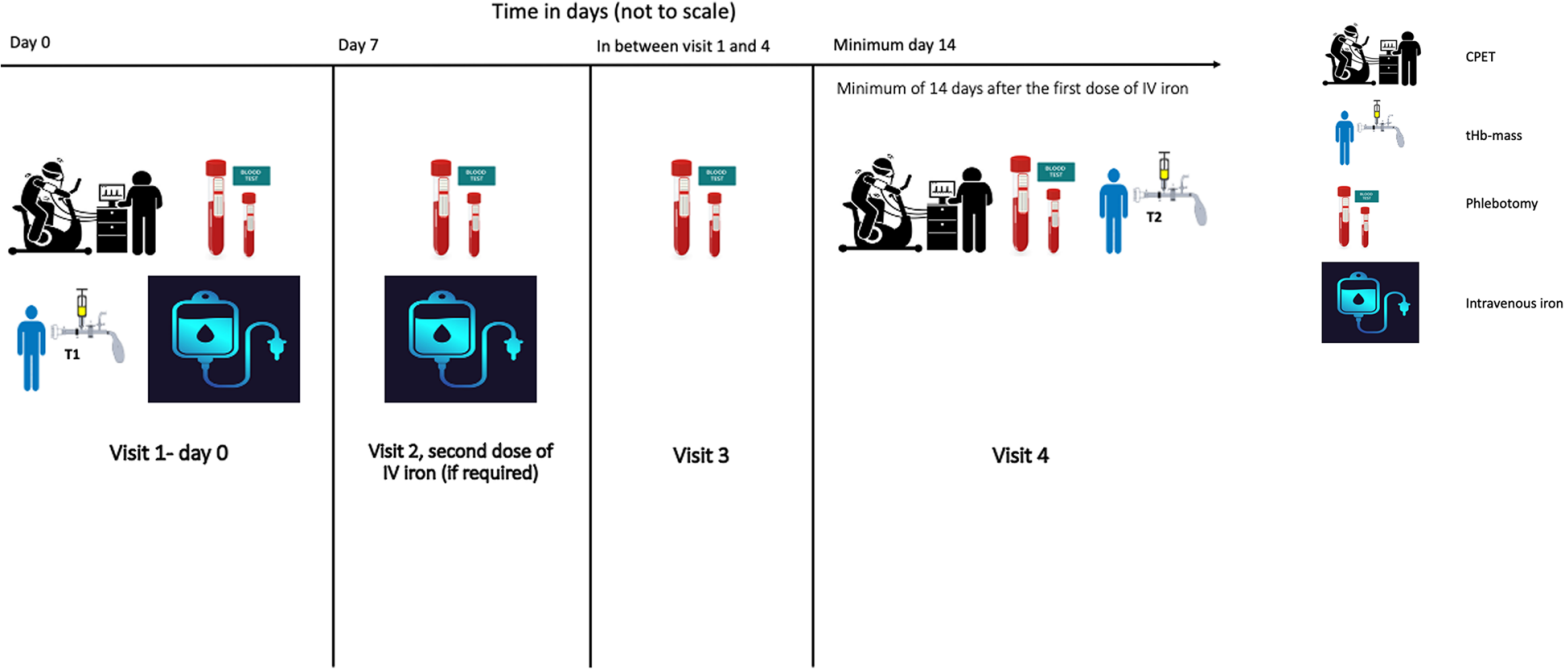
Study pathway

**Fig. 2:**
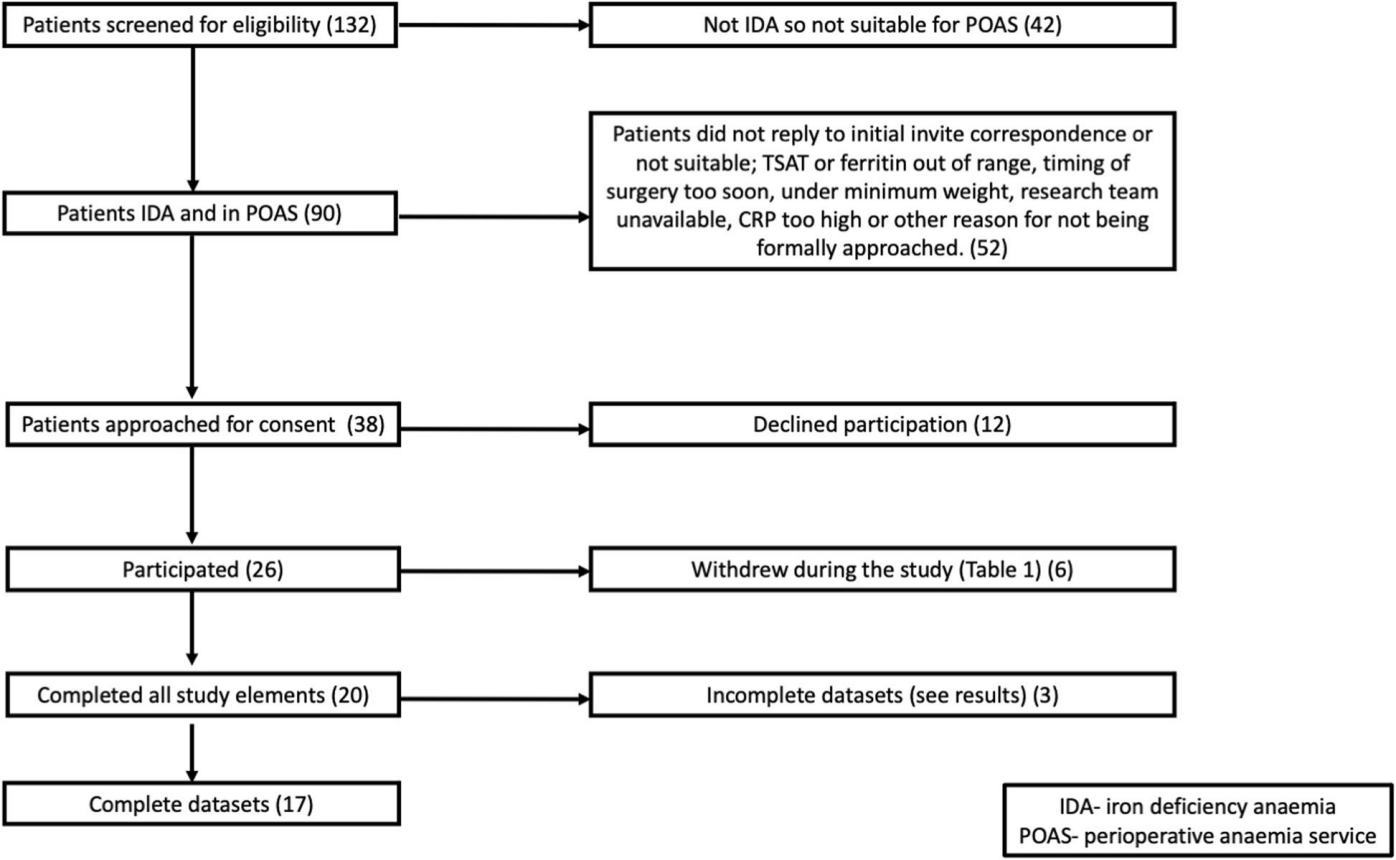
CONSORT diagram. CRP, C-reactive protein; IDA, iron deficiency anaemia; POAS, perioperative anaemia service; TSAT, transferrin saturation

Patients were recruited via clinical surgical teams, from the perioperative optimisation of anaemia before surgery (POAS) service or the clinical CPET service. Excluded were pregnant women; prisoners; those with known allergy/hypersensitivity to ferric derisomalotise (FDI) (also known as iron isomaltoside 1000) or any of its excipients, or to any parenteral iron products; those suffering from haemochromatosis or other iron overload states, acute liver or renal failure, active infection or haemoglobinopathies (e.g. sickle cell anaemia or thalassemia); those with other causes of anaemia (e.g. haematological malignancy, haemolysis, hypothyroidism); those receiving a blood transfusion before a second CPET could be performed; and those unable to perform CPET or in whom such testing was contraindicated (Additional file 1: Appendix 3 in Supplementary Material). See Figs. 1 and 2 for the study CONSORT diagram, study pathway (see Fig. 1).

After consent, patients underwent a baseline CPET and assessment of tHb-mass in addition to a standard panel of blood tests (Additional file 1: Appendix 4 in Supplementary Material) including measurement of iron indices (serum iron, transferrin and transferrin saturation). They then received dose 1 of intravenous (i.v.) iron. Iron dosing was based on the UHS POAS protocol (Additional file 1: Appendix 3 in Supplementary Material). Ferric derisomaltose, a high dose i.v. iron preparation, was used and was dosed according to the simplified dosing table described in the European Medicines Agency Summary of product characteristics (SmPC). Where a second dose was required to replenish total iron need, this was given exactly 1 week after the initial infusion. All infusions were undertaken in the Welcome Trust Clinical Research Facility at UHS and were overseen by the lead investigator. Participants came back a further 2 times (spread out once surgery date was known) for repeat blood tests before their final visit (4th visit a minimum of 10 days later) to repeat CPET and tHb-mass measurements (always in that order due to the small risk that CO gas might worsen CPET performance). The study period ended at the end of the final visit (see Fig. 1). Patients were informed to stop any oral iron supplements for the duration of the study period. Diet and exercise levels were not controlled in anyway.

Importantly, due to the known effect of exercise on haemoglobin concentration, blood was drawn prior to CPET testing for the baseline haemoglobin concentration. Subsequent blood was drawn after the rest periods described below for the purpose of tHb-mass measurement.

### Cardiopulmonary exercise testing

Firstly, an intravenous cannula was inserted into the subject’s upper limb and blood was taken for haemoglobin concentration and haematocrit measurements. Patients cycled on an electromagnetically braked ergometer (Ergoline 2000, Ergoline GmbH, Bitz, Baden-Württemberg, Germany). Respiratory gas analysis was performed using calibrated metabolic carts (Geratherm Respiratory GmbH; Love Medical Ltd, Manchester, UK). Breath-by-breath $\dot{\text{V}}$ O_2_ and carbon dioxide output (CO_2_) were recorded, concurrently with minute ventilation, tidal volume, respiratory rate and end-tidal gas tensions for O_2_ and CO_2_. Patients were connected to appropriate monitoring equipment and rested for an initial 3-min period, thereafter, completing 3 min of unloaded cycling. Subsequently, patients performed a symptom-limited incremental ramp test set to 10–20 W^.^min^−1^ (based on patient weight, and age allowing adjustment for clinical status and current activity levels) to deliver an intended test duration of 8–12 min before volitional exhaustion. Test cessation occurred at patient exhaustion or when the cadence reduced below 40 r.p.m. for more than 30 s despite verbal encouragement. After stopping CPET, patients completed a period of unloaded cycling to ‘cool down’.

The anaerobic threshold ($\dot{\text{V}}$ O_2AT_ expressed in millilitres of oxygen per kilogram per minute, ml^.^kg^.−1^ min^−1^) was determined by a clinical exercise physiologist and consultant physician (independently of each other) both skilled in CPET interpretation, using the modified V-slope method with corroboration by ventilatory equivalents and end-tidal gas tensions for O_2_ and CO_2_ (Bartoszko et al. 2019). The average $\dot{\text{V}}$ O_2_ throughout the final 20 s of exercise was recorded as the $\dot{\text{V}}$ O_2 peak_ (ml^.^kg^.−1^ min^−1^) (Levett et al. 2018). The patients were then rested prior to measuring tHb-mass. Patients were rested for 15 min before any blood was sampled and rested for a further 15 min prior to the start of the oCOR.

### Determination of tHb-mass, plasma and blood volumes

Subjects completed a baseline optimised carbon monoxide rebreathing test (oCOR, detailed in Additionals 1 & 2 of the Supplementary Material). Subjects were seated and inactive for 15 min before inhaling 0.8–1 ml^.^kg^−1^ of carbon monoxide (CO) mixed with 3 l of 100% oxygen via a glass spirometer (BloodTec, Bayreuth, Germany), which was rebreathed (via a CO_2_ scrubber) for 2 min whilst wearing a nose clip. A portable CO gas detector (Dräger Pac 7000, Drägerwerk AG & Co. KGaA, Germany) was used to detect possible CO leakage at the nose, mouthpiece and spirometer during rebreathing.

Carboxyhaemoglobin percentage was determined in venous blood samples drawn into Na-heparinised syringes (RAPIDLyte, Siemens Healthcare Diagnostics Inc., USA) before and at 6 and 8 min after administration of CO gas (analysis using a laboratory blood gas analyser; Radiometer, ABL800 FLEX). Each sample was analysed three times within 1 h of collection. The analyser was subjected to regular maintenance and quality control checks; the accuracy of which has been evaluated elsewhere (Turner et al. 2014). [Hb] and haematocrit values were measured using HemoCue (HemoCue AB, Radiometer, Sweden) and the blood gas analyser (Radiometer, ABL800 FLEX, Copenhagen), respectively.

All procedures were carried out by the lead investigator (JOMP) with assistance from SB.

### Statistical analysis

Statistical analysis was performed using GraphPad Prism (version 8.4.2c for Apple Macintosh OSX 10.14.4) and SPSS Statistics (version 25 for Apple Macintosh Chicago, IL, USA). The D’Agostino & Pearson normality test (omnibus K^2^ test) for normal distribution was used. Paired *t* tests were used to compare variables measured before and after intravenous iron if normally distributed, and the Wilcoxon signed-rank test was used where variables were not normally distributed. All tests were two-sided with a significance level of 0.05. Spearman’s rank was used to correlate relationships where data was non-parametric (CRP, tHb-mass and [Hb]). Values are presented as mean ± standard deviation (SD), unless otherwise stated. Median and interquartile range (IQR) are reported when variables were not normally distributed. Categorical variables are presented as frequency (%).

We based our sample size on previous studies augmenting [Hb] with allogenic blood transfusion. We were aware of 5 such studies in clinical subjects; 4 in Thalassemia patients that recruited 12 (Villa et al. 1996), 22 (Marinov et al. 2008), 13 (Grant et al. 2012) and 18 (Benedetto et al. 2015) patients respectively and one in patients with chronic anaemia (not from malignancy) which recruited 20 patients. The study of Wright et al. demonstrated an increase in $\dot{\text{V}}$ O_2 AT_ of 0.74 ml^.^kg^.−1^ min^−1^, and we based out power calculation upon this. G*Power was used for a priori power calculation which informed us that for a 0.7 ml^.^kg^.−1^ min^−1^ change in $\dot{\text{V}}$ O_2 AT_, we would need to recruit 19 patients using a alpha error of 0.05 and a beta error of 0.8 (Wright et al. 2014). We therefore recruited 26 patients to allow for dropouts.

Regarding the oCOR the typical error expressed as a coefficient of variation with 95% confidence limits (CL) has previously been published for the lead investigator 2% (1.67–2.59) (Krehl et al. 2021; Plumb et al. 2018) and is in keeping with previously published values (Siebenmann et al. 2017), 2.2% (1.4–3.5) (Gore et al. 2005). For cardiopulmonary exercise testing with the metabolic cart used in this study the quoted coefficient of variation (CV) is ± 3% (direct correspondence with Love Medical Ltd., Manchester, UK) which is in keeping with other published literature on metabolic carts.

The primary outcome measures were the changes in haematological variables—[Hb] (g^.^l^−1^) and [Hb] (g^.^l^−1^) and the changes in CPET variables $\dot{\text{V}}$ O_2 AT_ (ml^.^kg^.−1^ min^−1^) and $\dot{\text{V}}$ O_2_ peak (ml^.^kg^.−1^ min^−1^). The secondary outcome measures were split in haematological (changes in iron (mmol.l^−1^), transferrin (grams.l^−1^) and transferrin saturation (%)) and CPET derviced (Peak WR (watts) and Exercise time (seconds).

## Results

### Baseline characteristics and data loss

Twenty-six patients were recruited, of whom 6 withdrew prior to study completion (see Table 1). Of the remaining 20 patients who underwent all aspects of the study protocol, 1 had an uninterpretable tHb-mass test and 3 had aspects of their CPET that were uninterpretable (see Table 2). This left 17 with complete datasets, but all 20 patients are included in the final analysis, (see Tables 2 and 3), with a mean ± SD age of 68 ± 10 years, height 165 ± 9 cm, weight 76.5 ± 20 kg and BMI (median and [range]) 26.4 [23.6–30.7] kg^.^m^2^). There was a mean ± SD of 25 ± 7 days between baseline and the final testing visit (Table 2). The type of proposed surgery is displayed in Table 2.

**Table 1:** Study protocol deviations

Reason for non-completion	Number of patients
Surgery dates expedited preventing second CPET	**3**
Did not attend follow up (no reason given)	**1**
Admitted as an emergency due to rectal bleeding during the study and received a blood transfusion meaning they were ineligible to continue	**1**
Started but did not want to perform a repeat CPET	**1**

*CPET*, cardiopulmonary exercise test

**Table 2:** Individual patient demographics

SN	Patient ID	Gender	Age (years)	Height (cm)	Weight (kg)	BMI (kg^.^m^2^)	Days between Visit 1 and Visit 4	Type of surgery planned
1	1	M	55	186.6	95.7	27.5	13	Colorectal cancer
2	4	F	80	168.5	72.1	25.4	23	Colorectal cancer
3	6	M	79	168.7	83.7	29.4	20	Colorectal (non-cancer)
4	7	M	65	181.2	125.1	38.1	33	Orthopaedics
5	8	M	72	169	87.6	30.7	27	Colorectal cancer
6	9	F	69	161.2	71.0	27.3	29	Colorectal cancer
7	10	M	72	172.8	76.6	25.7	28	Colorectal cancer
8	12	F	49	146.4	53.2	24.8	21	Colorectal (non-cancer)
9	13	M	82	171.4	78.3	26.7	22	Colorectal cancer
10	14	M	75	158.5	59	23.5	42	Upper gastrointestinal cancer
11	15	M	83	170	64.2	22.2	24	Colorectal cancer
12	16	M	73	169	87.1	30.5	22	Colorectal cancer
13	17	F	80	157.3	47.1	19	33	Colorectal cancer
14	18	F	65	158.3	114	45.5	34	Colorectal cancer
15	19	F	66	156.5	63.7	26	14	Colorectal cancer
16	20	F	66	168.5	68	24	14	Hepatopancreaticobiliary cancer
17	21	F	55	159.1	55.3	21.8	21	Colorectal cancer
18	22	F	65	155	78.5	32.7	25	Spinal
19	25	F	51	167	92	33	24	Colorectal cancer
20	26	F	74	162.1	57.2	21.8	24	Colorectal (non-cancer)

*SN* study number

**Table 3:** Individual patient data from cardiopulmonary exercise testing, total haemoglobin mass and haemoglobin concentration

SN	Pre-$\dot{\text{V}}$ O_2AT_	Post-$\dot{\text{V}}$ O_2AT_	Δ $\dot{\text{V}}$ O_2AT_	Pre-$\dot{\text{V}}$ O_2_peak	Post-$\dot{\text{V}}$ O_2_peak	Δ $\dot{\text{V}}$ O_2_peak	CRP	Pre-[Hb]	Post-[Hb]	Δ[Hb]	Pre-tHb-mass	Post-tHb-mass	**ΔtHb-mass**
1	9.8	12.6	2.8	19.2	22.8	3.6	*No data*	91	100	9	*No data*	*No data*	*No data*
2	9.5	10.5	1	15.4	15.5	0.1	3	112	124	12	622	756	134
3	7	7	0	*No data*	*No data*	*No data*	16	123	129	6	592	640	48
4	6.2	7.3	1.1	10.8	12.3	1.5	3	113	119	6	729	783	54
5	7.2	9.7	2.5	14.4	15.6	1.2	23	126	123	− 3	695	700	4.8
6	*No data*	*No data*	*No data*	5.4	6.6	1.2	14	94	106	12	345	395	50
7	7.9	8.5	0.6	15.5	17.8	2.3	16	114	128	14	597	644	47.1
8	10.9	12.1	1.2	18.5	16.9	− 1.6	1	95	108	13	271	352	81.1
9	9.9	7.7	− 2.2	12.6	10.6	− 2	13	102	109	7	580	679	99.3
10	10.3	10.2	− 0.1	13.5	16.9	3.4	9	83	89	6	264	288	24.3
11	11.2	13.1	1.9	20.1	20.5	0.4	9	108	119	11	460	491	31
12	*No data*	*No data*	*No data*	*No data*	*No data*	*No data*	87	87	93	6	411	413	2.2
13	9.7	10.1	0.4	15.6	16.7	1.1	1	109	110	1	431	424	-6.8
14	8	7.6	− 0.4	11.6	12.6	1	10	125	128	3	608	678	70
15	9.3	8.6	− 0.7	17.8	18.6	0.8	1	114	122	8	450	515	65
16	10.9	11.3	0.4	21.1	20.6	− 0.5	3	116	117	1	527	554	26.5
17	11.6	15.8	4.2	20.3	23.7	3.4	11	90	114	24	368	478	110.2
18	10.4	9.8	− 0.6	14.1	15.1	1	8	125	125	0	447	493	45.7
19	7.5	7.4	− 0.1	*No data*	*No data*	*No data*	3	123	128	5	605	577	-28
20	7.2	6.5	− 0.7	13.1	13.6	0.5	6	122	127	5	443	521	78

*CRP* C-reactive protein (mg^.^l^−1^), *[Hb]* haemoglobin concentration (g^.^l^−1^), $\dot{\text{V}}$*O*_*2AT*_ oxygen consumption at anaerobic threshold (ml^.^kg^.−1^ min^−1^), $\dot{\text{V}}$*O*_*2*_* peak* peak oxygen consumption (ml^.^kg^.−1^ min^−1^), *tHb-mass* total haemoglobin mass (grams), *Δ* change pre and post iron supplementation, *SN* study number

Of the 20 patients analysed 15 went on to have an operation. Their length of hospital stay was a (median [IQR]) of 9 days [5–16]. Table 2 contains the surgical subspeciality with 80% being colorectal and 65% having colorectal cancer.

### Safety

No serious adverse events (SAEs) occurred during the study related to either CPET or i.v. iron infusion.

### Haematological variables

**Fig. 3:**
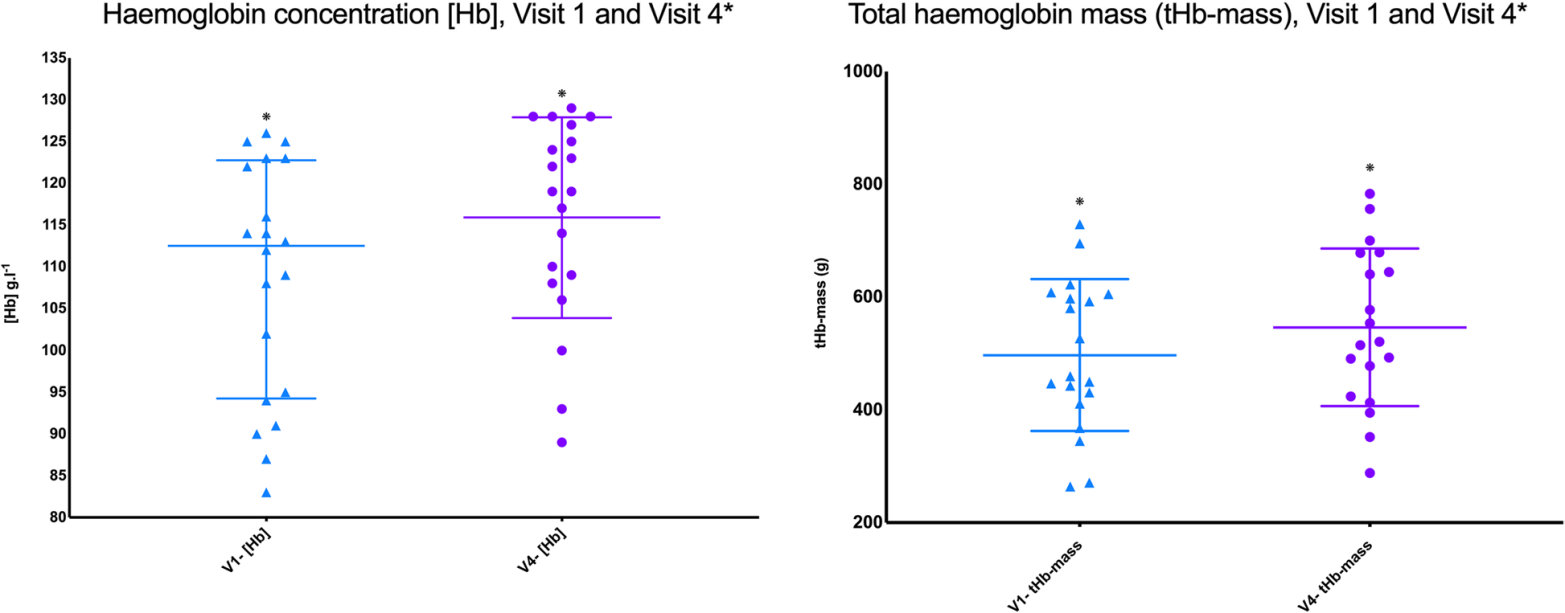
[Hb] and tHb-mass

Fifteen patients were iron-deficient based on a ferritin of 30 ng.ml^−1^ or less. Four patients had functional iron deficiency based on a ferritin > 30 ng.ml^−1^ but a TSAT < 20% (Additional file 1: Appendix 3 in Supplementary Material). The one patient who fell slightly outside of the criteria set in Additional file 1: Appendix 3 had a ferritin of 52 ng.ml^−1^ and a TSAT of 23% and was therefore arguably iron deplete (Plumb et al. 2016a). Following administration of i.v. iron, both [Hb] and tHb-mass rose in all but 4 patients (subjects 5 and 18 for [Hb] and subjects 13 & 19 for tHb-mass: see Tables 3 and 4 and Fig. 3, respectively. [Hb] increased from (mean ± SD) 109 ± 14 g^.^l^−1^ to 116 ± 12 g^.^l^−1^ (6.4% rise: mean difference of 7.3 ± 6 g^.^l^−1^, *p* =  < 0.0001, 95% CI 4.5–10.1). tHb-mass rose from 497 ± 134 g to 546 ± 139 g (9.8% rise: mean difference 49 g, *p* =  < 0.0001, 95% CI 29.4–69.2). Similarly, serum iron concentration rose from 7.2 ± 4.3 to 12.3 ± 4.2 mmol.l^−1^ (*p* < 0.0001, 95% CI 2.9–7.3) and transferrin saturation from 13.1 ± 8.5 to 25.3 ± 7.6% (*p* 0.0002, 95% CI 6.7–17.8) with transferrin concentration decreasing from 3.0 [2.8–3.3] to 2.0 [1.3–2.4] g^.^l^−1^ (*p* < 0.0001) (see Table 4).

**Table 4:** Haematological and cardiopulmonary exercise testing variables pre- and post-intravenous iron

**Variable**	**Baseline**	**Final visit**	Δ	**Statistical test**	***p*** ** value**
[Hb] (g^.^l^−1^)	109 ± 14	116 ± 12	7.3 ± 6.4	Paired *t* test	< 0.0001
tHb-mass (grams)	497 ± 134.0	546 ± 139	49.3 g ± 41.3	Paired *t* test	< 0.0001
Iron (µmol.l^−1^)	7.2 ± 4.3	12.3 ± 4.2	6.3 ± 3.5	Paired *t* test	< 0.0001
Transferrin (grams.l^−1^)	3.0 [2.8–3.3]	2.0 [1.3–2.4]	0.8 [0.4–1.1]	Wilcoxon	< 0.0001
Transferrin saturation (%)	13.1 ± 8.5	25.3 ± 7.6	16.2 ± 6.2	Paired *t* test	0.0002
$\dot{\text{V}}$ O_2 AT_ (ml^.^kg^.−1^ min^−1^)	9.1 ± 1.7	9.8 ± 2.5	0.63 ± 1.51	Paired *t* test	0.0964
$\dot{\text{V}}$ O_2_ peak (ml^.^kg^.−1^ min^−1^)	15.2 ± 4.1	16.3 ± 4.4	1.0 ± 1.6	Paired *t* test	0.0165
Peak WR (watts)	93 [67–112]	96 [68–122]	5 [-2–13]	Wilcoxon	0.0198
Exercise time (seconds)	475 ± 130	493 ± 140	17.7 ± 58.7	Paired *t* test	0.2041

*[Hb]* haemoglobin concentration, $\dot{\text{V}}$*O*_*2AT*_ oxygen consumption at anaerobic threshold, $\dot{\text{V}}$*O*_*2*_* peak* peak oxygen consumption, *Peak WR* peak work rate, *tHb-mass* total haemoglobin mass, *Δ* change pre and post iron supplementation. Data reported as mean ± SD or median [range]

C-reactive protein (CRP) (median [IQR]) in the period of testing (highest value taken pre-operation or during study period (if no operation performed) was 9 [3–14] mg.l^−1^. One patient had a CRP of 87 mg.l^−1^ (Table 3). There was no correlation between change in [Hb] and CRP (*r* 0.06, *p* 0.8) or change in tHb-mass and CRP (*r − *0.11,* p* 0.6). The mean difference between recruitment [Hb] and [Hb] on test day 1 was 0.6 g^.^l^−1^ (CI − 2.0–3.2) with a *p* value of 0.63.

### Cardiopulmonary exercise testing variables

**Fig. 4:**
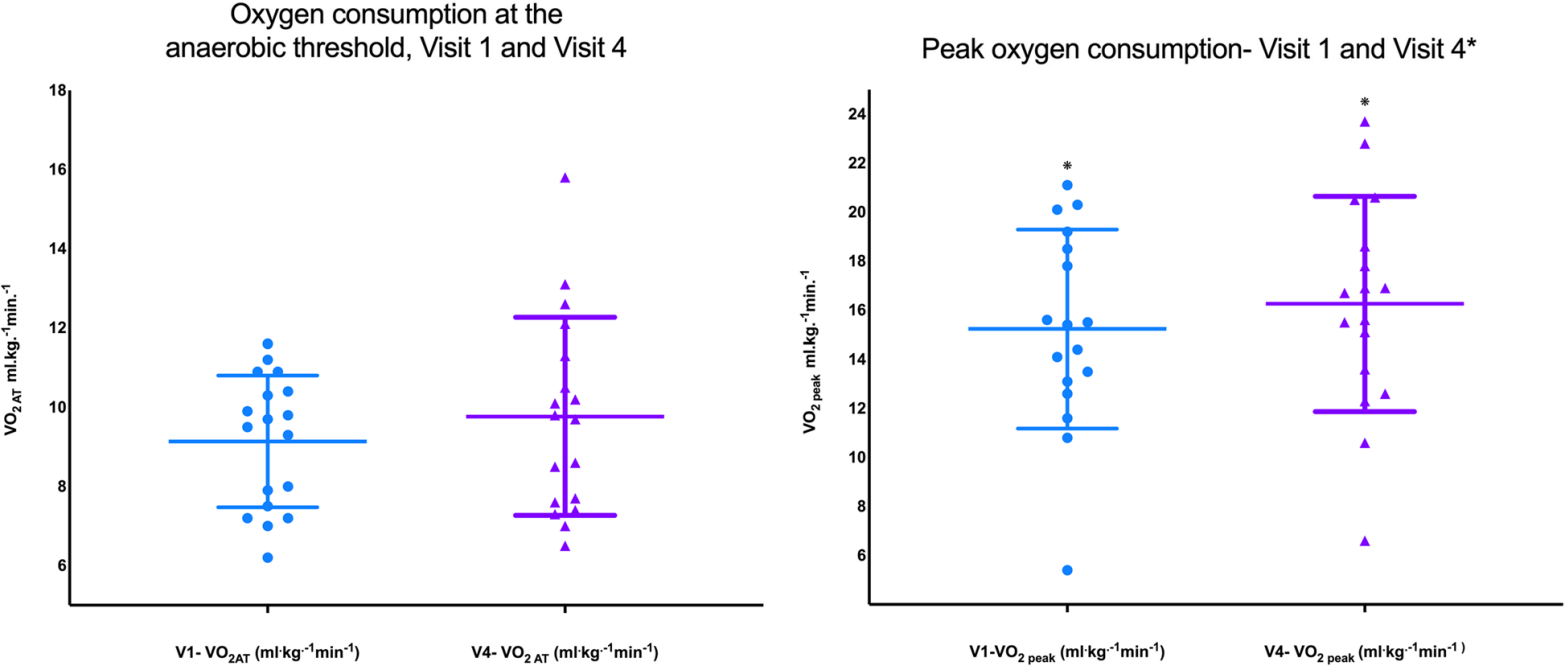
CPET variables

Mean ± SD $\dot{\text{V}}$ O_2 AT_ was 9.1 ± 1.7 ml^.^kg^.−1^ min^−1^ at baseline and 9.8 ± 2.5 ml^.^kg^.−1^ min^−1^ after i.v. iron (mean difference of 0.63 ± 1.5 ml^.^kg^.−1^ min^−1^, *p* = 0.09). $\dot{\text{V}}$ O_2 peak_ rose from 15.2 ± 4.0 to 16.3 ± 4.4 ml^.^kg^.−1^ min^−1^ (mean difference 1.0 ± 1.6 ml^.^kg^.−1^ min^−1^, *p* = 0.02) (see Fig. 4). Peak work rate rose from 93 [67–112] watts to 96 [68–122] watts (*p* = 0.02). The mean ± SD ramped exercise time was 475 ± 130 s at baseline and 493 ± 140 s at final testing visit (*p* = 0.58).

## Discussion

To our knowledge, this is the first study to demonstrate that administration of i.v. iron to iron-deficient/deplete perioperative patients with anaemia was associated with increases in $\dot{\text{V}}$ O_2 peak_ and peak work rate, along with previously observed increases in [Hb] and tHb-mass.

Importantly, this was achieved without disruption to a patient’s normal perioperative care pathway. Of note, 5 patients did not go on to have surgery. This is because in our pathway, we identify patients very early, often at the point of presumed diagnosis (for example, a positive lower GI endoscopy but without a tissue diagnosis) prior to a multi-disciplinary meeting and aim to treat their anaemia regardless of whether they go on to undergo surgical resection.

The rise in [Hb] after i.v. iron is in keeping with other perioperative studies using intravenous iron to treat anaemia (Froessler et al. 2016; Keeler et al. 2017; Kim et al. 2017; Moppett et al. 2019; Xu et al. 2019). UK national guidance (NICE 2016) and international consensus (Muñoz et al. 2017) on the management of perioperative anaemia and iron deficiency support the use of intravenous (i.v.) iron (if oral iron is ‘not appropriate’ or planned surgery is < 6 weeks away) as an alternative to blood transfusion, albeit that data relating to the efficacy of this approach are equivocal (Froessler et al. 2016; Keeler et al. 2017, 2014; Moppett et al. 2019).

The PREVENTT study (Richards et al. 2020) found a modest increase in haemoglobin ([Hb]) of 4.7 g/l following a 1000-mg dose of ferric carboxymaltose (Ferrinject®) compared to placebo. However, it is important to consider that not all patients in the PREVENTT study had iron-deficiency anemia. Only 76% had transferrin saturation (TSAT) levels below 20%, and 57% had ferritin levels below 100 ng/ml, whereas in our study, these percentages were 89% and 100%, respectively.

Additionally, the median time from treatment to surgery differed between the PREVENTT study and our current study. In the PREVENTT study, the median time was 14 days (range 5–212 days) after intravenous (i.v.) iron administration, while in our study, it was 25 days (range 13–42 days). This extended time window in our study may have allowed for a greater increase in total haemoglobin mass (tHb-mass) and [Hb] levels.

It is worth noting that the full treatment effect of i.v. iron preparations typically takes around 6 weeks (Bhandal and Russell 2006; Goodnough et al. 2009). However, some studies have observed a haematopoietic effect as early as 5 days (Johansson et al. 2015). For example, in an RCT involving inflammatory bowel disease (IBD) patients (Evstatiev et al. 2011), a significant increase in [Hb] was observed at 14 days following intravenous iron administration, and this improvement continued up to 8 weeks. Similarly, another recent RCT comparing ferric derisomaltose with iron sucrose reported a mean change in [Hb] of 15 g/l at 14 days post-treatment, which further increased to approximately 25 g/l at 8 weeks (Auerbach et al. 2019). While we acknowledge that achieving longer lead times may be challenging for other centers, the advantages of converting patients from a ‘waiting list’ to a ‘preparation list’ have been described by The Centre for Perioperative Care (CPOC) in the UK. It is important to recognize that different countries may have varying practices, and attaining a preparation time of 6–8 weeks may not always be feasible on an international scale.

Inflammation levels were generally low (median CRP: 9 mg/l), possibly resulting in negligible changes in [Hb] or tHb-mass for a few patients (5 and 12, see Table 3). Inflammatory-driven alterations in hepcidin levels can hinder oral iron absorption. However, evidence suggests that i.v. iron can be effective even in patients with elevated CRP. In the IRONMAN trial, the median [Hb] was 110 g.l^−1^ [48–170], and interestingly, CRP did not correlate with hepcidin levels (Litton et al. 2018).

To maintain the routine care pathway, we set a minimum of 10 days between iron administration and repeat CPET. However, we actually waited 13 days (Table 2), a timeframe in which significant increases in [Hb] have been previously observed (Bhandal and Russell 2006; Goodnough et al. 2009). The modest rise of 7.3 g.l^−1^ in [Hb] and 49 g in tHb-mass suggests that we may not have measured at the optimal treatment response time. Given a longer interval between repeated CPETs, further increases in both [Hb] and tHb-mass are likely. Nevertheless, our observed [Hb] increase aligns with other preoperative studies using i.v. iron (8 g.l^−1^ (Froessler et al. 2016), 15.5 g.l^−1^ (Keeler et al. 2017); 8 g.l^−1^), (Khalafallah 2012) and 9 g g.l^−1^ (Khalafallah et al. 2015), although it is lower than some studies (Diez-Lobo et al. 2007) 22 g.l^−1^. This lesser impact could be attributed to ongoing tumour-related blood loss.To our knowledge, this is the first time that tHb-mass has been measured in perioperative patients after receiving intravenous iron. We observed a mean increase in tHb-mass of 49 g. Unfortunately, what constitutes a ‘normal’ or clinically meaningful rise in an anaemic subject remains to be defined. Nonetheless, we have previously described and demonstrated that the relationship of tHb-mass with perioperative performance is greater than that seen with [Hb] (Otto et al. 2017a; Otto et al. 2017b; Plumb et al. 2020; Plumb et al. 2018; Plumb et al. 2016b).

Where [Hb] or tHb-mass did not increase post i.v iron, the differences were within the laboratory test variation for the respective variables. Table 3 documents the individual patients in whom this was applicable (patients 5, 13, 18 & 19). Patient 5 had only borderline iron deficiency anaemia and therefore perhaps unsurprisingly went from [Hb] 126 to 123 g^.^l^−1^ and tHb-mass from 695 to 700 g. The other 3 patients had small variations within laboratory normal ranges so could represent technical error, non-response to i.v. iron or perhaps greater Hb loss may have been occurring in such subjects.

Importantly, tHb-mass is not affected by day to day changes in plasma volume or by acute exercise and is not subject to the known fluctuations that haemoglobin concentration is (Garvican et al. 2010a; Garvican et al. 2010b; Schumacher et al. 2008). This has been demonstrated in a cohort of cyclists at altitude, where in native altitude dwellers, tHb-mass did not change during a cycle stage race at altitude, but in low land natives, it increased. In the same group of low land natives, tHb-mass did not change in an equivalent event at sea level demonstrating that the altitude was responsible rather than exercise over a short time period (Garvican-Lewis et al. 2014). Haemoglobin concentration is affected by endurance exercise due to plasma volume adaptation to prolonged exercise. Data exits from small studies to support acute changes in [Hb]; however, it returns to normal within a short time frame (Gwozdzinski et al. 2013; Schumacher et al. 2010).

### Cardiopulmonary exercise testing variables

$\dot{\text{V}}$ O_2 peak_ and peak work rate rose following i.v. iron (*p* = 0.02 in both cases), findings which *may* have clinical significance and which are hypothesis generating with regard to i.v. iron improving haemoglobin mass and resulting in greater physiological fitness and possibly resilience to surgery. Improvements in $\dot{\text{V}}$ O_2 peak_ have been demonstrated in chronic heart failure alongside improvements in functional status (NYHA classification) following treatment with i.v. iron (Okonko et al. 2008), while others have shown improvements in 6-min walk test distance (6MWD) following i.v. iron therapy (Anker et al. 2009; Ponikowski et al. 2015). Similar findings in 6MWD performance have been observed in patients with pulmonary hypertension following i.v. iron (Viethen et al. 2014), although a postoperative study comparing i.v. and oral iron in patients after total knee arthroplasty found no difference in 6MWD (Bisbe et al. 2014).

Observational data suggest that exercise capacity is affected by the extent of iron deficiency (Jankowska et al. 2011), making it potentially modifiable. For instance, the EFFECT-HF study in patients with congestive heart failure and iron deficiency demonstrated improved $\dot{\text{V}}$ O_2 peak_ with i.v. ferric carboxymaltose compared to standard care, regardless of anaemia status (Van Veldhuisen et al. 2017). However, this effect was highly sensitive to the imputation strategy used for $\dot{\text{V}}$ O_2 peak_ among patients who died and so remains to be further assessed.

In the perioperative setting, a recent pre-specified sub study of the Measurement of Exercise Tolerance before Surgery (METS) study demonstrated that anaemia (defined using [Hb]) explained only 3.8% of the variation in $\dot{\text{V}}$ O_2 peak_ in a multi-variate regression model (Bartoszko et al. 2019). We have shown similarly low explained variance of [Hb] in $\dot{\text{V}}$ O_2 peak_ (9%) and $\dot{\text{V}}$ O_2 AT_ (6%), highlighting that other factors may play a key role as fitness determinants, with tHb-mass being one such important candidate (Otto et al. 2013). It is also possible that i.v. iron improves exercise capacity via improvements in mitochondrial function in the skeletal muscle via improvements in phosphocreatine generation (Charles-Edwards et al. 2019).

Elevating tHb-mass in elite athletes is consistently associated with a proportional increase in $\dot{\text{V}}$ O_2 max_ (Saunders et al. 2013) with such increases being underpinned by changes in arterial oxygen content and systemic oxygen transport. Early animal data is suggestive of iron improving exercise performance (Finch et al. 1976). However, there are mixed results in athletes with regard to iron improving performance with some studies supporting improved performance (Friedmann et al. 2001) (oral iron) (Garvican et al. 2014) (i.v. iron) and others demonstrating no changes in CPET performance (Burden et al. 2015b; Peeling et al. 2007) (i.v. iron). A meta-analysis of iron supplementation in non-anaemic iron deficient athletes (IDNA) concluded that exercise performance is increased with iron therapy (Burden et al. 2015a).

Previous work in anaemic patients receiving blood transfusions has demonstrated increases in CPET variables, but these data are complicated by the known significant effect of blood volume increases on those same variables aside from any effects of the increased O_2_ carrying capacity that a transfusion affords (Wright et al. 2014). Numerous historical studies in elite athletes have examined the relationship between allogenic blood transfusions (Sawka et al. 1996) and exercise variables, which we have previously reviewed elsewhere (Plumb et al. 2016b). Interestingly, many of these although showing some improvement in exercise performance were underpowered and debate remains around the relative contributing factors with regard to improved O_2_ delivery in these circumstances, namely the blood volume vs. the haemoglobin mass changes.

The heterogenous nature of the cohort (different surgical specialities) limits our ability to perform sub-group analysis for anaemia severity and impact on exercise performance. Another potential weakness was the time between i.v. iron and repeat CPET not being long enough for the full treatment effect to be observed (see above). What does this study tell us?

Administration of i.v. iron to iron-deficient preoperative patients with mild anaemia is associated with increases in [Hb], tHb-mass and $\dot{\text{V}}$ O_2 peak_.

### Unanswered questions and future research

In theory, anaerobic threshold might rise after i.v. iron due to increased physical activity related to improved aerobic capacity (a training effect). Alternatively, impacts of iron deficiency on the enzymes of the electron transport chain might be mitigated (Haddad et al. 2017; Hamano 2019). Whilst anaerobic threshold did not change significantly in our study, this might have related to underpowering; post hoc analysis suggests that 34 subjects would be required with an alpha error of 0.05 and a beta error of 0.8 for a significant change in the anaerobic threshold. Further appropriately powered studies are required to examine the effect of intravenous iron upon exercise variables in a preoperative setting. In addition, the utility of alternative physiological end points may be more appropriate to guide and assess interventional changes following anaemia and/or iron optimisation. For example, an incremental ramped exercise test to symptom limitation may not be sensitive enough to evaluate the efficacy of some interventions and does not reflect activities of daily life which are in general sub-maximal and continuous in nature. Therefore, the use of constant work rate tests at a certain percentage of the maximal work rate achieved on an incremental exercise test may be more appropriate to assess exercise tolerance and more sensitive to changes in fitness than $\dot{\text{V}}$ O_2 peak_ or $\dot{\text{V}}$ O_2 AT_. These sorts of tests should be included in any future studies in this area (Barberan-Garcia et al. 2018; Puente-Maestu et al. 2016). In addition, wellbeing factors are also important and potential candidate mechanisms for i.v. iron and future work should include a robust quality of life analysis. Ultimately, the question that remains elusive is whether improved exercise performance via improvement in any haematological domain (be it red cell mass or otherwise) improves perioperative outcomes. This should be the focus of future research in this area.

## Conclusion

Preoperative administration of intravenous iron to iron-deficient/deplete anaemic patients is associated with increases in [Hb], tHb-mass, peak exertional oxygen consumption and peak work rate. Further appropriately powered prospective studies are required to ascertain whether improvements in tHb-mass and performance in turn lead to reductions in perioperative morbidity.

## Supplementary Information

**Additional file 1.**

## Data Availability

Reasonable requests for original data and protocols can be made in writing to the corresponding author.

## Abbreviations

AT: Anaerobic threshold
BMI: Body mass index
CI: Confidence interval
CL: Confidence limits
CO: Carbon monoxide
CO_2_: Carbon dioxide output
CPET: Cardiopulmonary exercise testing
CRP: C-reactive protein
CV: Coefficient of variation
FDI: Ferric derisomalotise
[Hb]: Haemoglobin concentration
IBD: Inflammatory bowel disease
IDNA: Non-anaemic iron deficient
IDA: Iron deficiency anaemia
IQR: Interquartile range
i.v: Intravenous
METS: Measurement of Exercise Tolerance before Surgery
oCOR: Optimised carbon monoxide rebreathing
O_2_: Oxygen
Peak WR: Peak work rate
POAS: Perioperative anaemia service
PREVENTT: Preoperative intravenous iron to treat anaemia in major surgery
PV: Plasma volume
6MWD: Six minute walk test distance
RCT: Randomised controlled trial
SAE: Serious adverse event
SD: Standard deviation
SmPC: Summary of produce characteristics
SN: Study number
tHb-mass: Total haemoglobin mass
TSAT: Transferrin saturation
UHS: University Hospital Southampton
$$\dot{\text{V}}$$ O_2_: Oxygen consumption
$\dot{\text{V}}$ O_2 AT_: Oxygen consumption at anaerobic threshold
$\dot{\text{V}}$ O_2 max_: Maximal oxygen consumption
$\dot{\text{V}}$ O_2 peak_: Peak oxygen consumption
WHO: World Health Organization
