# Supplementary material for: Cardiopulmonary exercise testing before and after intravenous iron in preoperative patients: a prospective clinical study
Source: Perioper Med (Lond). 2023 Jul 3;12:31. doi: 10.1186/s13741-023-00319-x (PMC10316643; doi:10.1186/s13741-023-00319-x)
Supplement: Supplementary file 1 — Additional file 1. [file 13741_2023_319_MOESM1_ESM.docx]

**Supplementary Material**

**Appendix 1- Calculation of tHb-mass**

tHb-mass was calculated using a specifically design excel spreadsheet (Microsoft Excel

2011 for Apple Macintosh) using the formula:

- tHb-mass (g) = K x MCO(ml) x 100 x (Δ%COHb x 1.39)^-1^
- K = barometric pressure x 760-1 x [1(0.003661 x temperature)]
- MCO = COadm – (COsystem + lung (after disconnection) + COexhaled (after disconnection)
- COadm = CO volume administered into the system
- COsystem + lung (after disconnection) = CO concentration in spirometer x (spirometer volume + remaining volume in the lung after disconnection)
- COexhaled (after disconnection) = end-tidal CO concentration x alveolar ventilation x time
- Δ%COHb = difference between baseline %COHb and %COHb post CO administration
- (average of 6- and 8-min %COHb values)
- 1.39 = H$\ddot{\text{u}}$fner’s number (constant) (ml CO x g Hb-1)

Residual volume and alveolar ventilation were calculated according to “Standardized lung function testing. Official statement of the European Respiratory Society” ^59^. CO concentration is in parts per million (ppm).

Blood volume (BV), plasma volume (PV) and red cell volume (RCV) were calculated

from mean corpuscular haemoglobin concentration (MCHC), [Hb] and tHb-mass, as

below:

- BV (ml) = tHb-mass (g)/[Hb] (g.dl^-1^) • 100
- RCV (ml) = tHb-mass (g)/MCHC • 100
- PV (ml) = BV – RCV

**Appendix 2- Measurement of tHb-mass using the Optimized Carbon Monoxide Re-Breathing Method (oCOR) and Typical Error measurement**

The use of CO to determine tHb-mass was first proposed in the late 1800s, with refined techniques being published 100 years later ^60^. In 2005, Schmidt and Prommer reported a simpler and faster technique (described in detail below), which also required less blood sampling ^61^. It was applied almost exclusively, however, in the fields of athletic physiology, and thus failed to come to the attention of the bulk of the broader clinical/medical community.

tHb-mass was determined using the validated oCOR method described in detail by Schmidt and Prommer ^61^. COHb concentration in blood was measured before and after 2 min rebreathing a known CO volume (0.5 to 1.0 ml^.^kg^−1^ in this study depending on sex and [Hb]). Each participant was seated for 15 min to allow stabilization of plasma volume (PV), after which a mouthpiece connected them *via* a container of ‘soda lime’~10g (carbon dioxide scrubber) to a spirometer (Spico-CO Respirations-Applicator, Blood Tec, Germany) and a 3-litre anesthetic bag pre-filled with 100% oxygen. The patient exhaled to residual volume, breathed in the CO dose *via* the spirometer, held their breath for 10s, then continued normal breathing into the closed circuit *via* the spirometer for 1 min 50s. The participant then exhaled to residual volume, this exhaled volume being collected and analyzed to quantify the CO not absorbed into the bloodstream. Disconnected from the mouthpiece, participants finally fully exhaled to residual volume into a CO gas analyzer (Dräger Pac 7000, Drägerwerk AG & Co. KGaA, Germany) before and at 4 min after CO rebreathing, in order to determine the end tidal CO concentration and thereby the amount of CO exhaled after disconnecting the patient from the spirometer, that will also have not been absorbed into the blood.

Typical error of measurement (TE) for tHb-mass was calculated and expressed as coefficient of variation with 95% confidence limits (CL), derived from χ^2^ distributions. All tests were 2-sided and statistical significance was set at *p*<0.05. TE includes random error (analytic error arising from using the method-specific apparatus and intra-individual biological variation) but not systematic error (bias) ^62–64^. Reported studies using the oCOR have commonly reported this method ^37 38 65 66^.

**Appendix 3 UHS protocol for i.v. iron (POAS)**

| Adult Monofer infusion guideline for patients undergoing surgery | | Version: | 2 |
| --- | --- | --- | --- |
| Date Issued: | March 2020 | | |
| Review Date: | March 2022 | | |
| Document Type: | Guideline | | |

| Contents |  | Page |
| --- | --- | --- |
| Paragraph | Executive Summary / Policy Statement / Flowchart | 2 |
| 1 | Scope and Purpose | 2 |
| 2 | Details of Procedure to be followed | 2 |
| 3 | Roles and Responsibilities | 2 |
| 4 | Related Trust Policies | 2 |
| 5 | Communication Plan | 2 |
| 6 | Process for Monitoring Compliance/Effectiveness | 2 |
| 7 | Arrangements for Review of this Policy | 2 |
| 8 | References | 2 |

| Appendices |  | Page |
| --- | --- | --- |
| Appendix A | Monofer Prescribing and Administration Guideline | 4 |
| Appendix B | Dose Calculation Flowchart | 6 |
| Appendix C | Grading and management of acute hypersensitivity reactions to intravenous iron infusions | 7 |

| Document Status |
| --- |
| This is a controlled document. Whilst this document may be printed, the electronic version posted on the intranet is the controlled copy. Any printed copies of this document are not controlled.  As a controlled document, this document should not be saved onto local or network drives but should always be accessed from the intranet. |

**Executive Summary**

Preoperative anaemia (defined by Hb<130g.l^-1^) increases perioperative morbidity and mortality in addition to increasing the transfusion risk. Intravenous iron is one of the therapies used in its management. All patients with preoperative anaemia should be referred back to primary care for investigation of the cause where appropriate.

Iron deficiency is normally treated satisfactorily by the use of oral iron supplements. However there are four circumstances when parenteral iron is recommended to replace iron stores, when; (i) oral iron supplements are tolerated poorly by some patients leading to non-adherence; (ii) there is insufficient absorption of iron from the gut due to an underlying illness such as renal disease or Crohn’s disease; (iii) the rate of iron loss exceeds the total absorption rate of iron from the gut; or (iv) surgery is planned <8 weeks and cannot be delayed due to urgency of the treatment. Those patients requiring intravenous iron are primarily seen as day case patients at Victoria House, however for the preoperative patients, they are treated on Surgical Day Unit, F level theatres recovery or on the surgical wards at Southampton General Hospital, or at Lymington Hospital.

1. **Scope and Purpose**

This guideline is relevant to patients undergoing surgery. It is aimed at doctors, pharmacists and nurses involved in the administration of Monofer to patients over 18 years of age.

1. **Details of Procedure to be followed**

The prescriber will be able to use this guideline, along with basic patient details, to calculate the appropriate Monofer® dose for an individual patient. This should be prescribed on the electronic prescribing system (for SGH/PAH site) and on paper non-EPMA prescription chart for Lymington Hospital. The infusion should be prepared and administered according to the prescription and the administration recorded.

1. **Roles and Responsibilities**

Medical staff

- - It is the admitting doctor’s responsibility to ensure that treatment with intravenous iron is appropriate and other causes of anaemia have been excluded (including monitoring liver function tests).
  - Review the contraindications and cautions to treatment. If any of these apply to the patient then there must be documentation of the risk-benefit balance.
  - It is the initiating doctors’ responsibility to ensure that the appropriate monitoring (including haemoglobin and serum ferritin) is performed, reviewed and patients followed up appropriately. The frequency of monitoring will need to be decided on a case-by-case basis.
  - It is the responsibility of the medical team to ensure that the iron infusion is prescribed.
  - It is the consultant’s responsibility to ensure that the guideline is followed and specialist advice requested if required.

Nursing staff

- It is the admitting nurse’s responsibility to weigh the patient (if not done within the last 6 weeks) to enable accurate calculation of the iron dose.
- Check for contraindications and cautions and ensure there has been proper consideration if any apply.
- Ensure that the infusion is prepared and administered safely and appropriately.
- The nursing staff will inform the patient about the risk of skin extravasation and discolouration. Patients will be asked to inform them immediately if they experience any pain at the cannula site.
- It is the responsibility of the discharging nurse to ensure that patients are supplied with verbal and written information about the treatment.

**Pharmacy Staff**

- It is the responsibility of the ward pharmacist to highlight any deviation from the guideline to the patient’s medical team.
- It is the responsibility of the surgical pharmacist or their supervisor to ensure these guidelines remain updated.

1. **Related Trust Policies**

Medicines – prescribing, acquisition, storage and administration.

1. **Communication Plan**

The guideline will be available on the Trust intranet.

1. **Consent**

Explain the procedure to the patient and gain verbal informed consent.

1. **Therapeutic indications in perioperative setting**

Monofer® is indicated for the treatment of iron deficiency in the following circumstances:

- When oral iron preparations are ineffective or cannot be used;
- Where there is a clinical need to delivery iron rapidly.

The diagnosis of iron deficiency must be based on appropriate laboratory tests (serum ferritin, transferrin saturation and haemoglobin).

1. **Process for Monitoring Compliance/Effectiveness**

There is prospective data collection and periodic analysis of safety and efficacy of Monofer® in perioperative care setting by the Perioperative Anaemia Service. Any areas of concern identified will be fed back to the appropriate clinicians.

1. **Arrangements for Review of the Policy**

Every three years.

1. **References**
2. Summary of Product Characteristics Monofer®. Available at [www.medicines.org.uk](http://www.medicines.org.uk) Last updated 15/10/2015 accessed February 2016
3. Personal Communication. Tim Greer. Pharmacosmos UK Ltd. 18/02/2016
4. ‘Intravenous iron for the treatment of pre-operative anaemia in adults’ Royal National Orthopaedic Hospital NHS Trust July 2018
5. ‘ A protocol for use and administration of intravenous iron isomaltoside (Monofer®)’ Barking, Havering and Redbridge University Hospitals NHS Trust July 2019
6. ‘Protocol for intravenous iron isomaltoside in the perioperative care setting for the treatment of iron deficiency anaemia where oral iron is not appropriate’ The Newcastle upon Tyne Hospitals NHS Foundation Trust January 2018
7. ‘Intravenous iron isomaltoside for correction of pre-operative iron deficiency anaemia in adults undergoing cardiac surgery’ Royal Papworh Hospital August 2018
8. ‘Guidelines for the use of parenteral iron in adults’ Royal United Hospitals Bath June 2017

**Appendix A – Clinical indications**

Monofer® therapy in preoperative patients is indicated in anaemic patients with absolute or functional iron deficiency.

Oral iron should be used as a first line treatment option where possible and is indicated in iron deficiency anaemia (IDA) where surgery is not urgent.

The diagnosis of iron deficiency must be based on laboratory tests.

The use of Monofer® in the preoperative setting is for patients with IDA who have one of the following and fit the criteria for the anaemia pathway:

- Undergoing major surgery where the interval between diagnosis of IDA and date of procedure is predicted to be short (time to surgery <8 weeks)
- IDA with history or poor inadequate response to oral iron therapy after eight weeks of treatment
- IDA with a history or poor compliance or intolerance to oral iron due to side effects
- Functional IDA
- Mixed functional and true iron deficiency anaemia

Patients with non-anaemic iron deficiency are considered as a case by case scenario. (for eg blood refusers, high risk patient, high risk surgery)

Anaemia: Hb<130g/L for both men and women

Iron deficiency anaemia: Ferritin <30 µg.l^-1^

Functional iron deficiency anaemia (FIDA): Ferritin>100 µg.l^-1^ + TSAT <20%

Mixed IDA/FIDA (Ferritin 30-100 + TSAT<20%)

**Appendix B – Monofer® Prescribing and Administration Guideline**

The purpose of this guideline is to advise medical and nursing staff on the prescribing and administration of Monofer. Please refer to the Summary of Product Characteristics (available at [www.medicines.org.uk](http://www.medicines.org.uk)) for full prescribing information.

Monofer® (iron (III) isomaltoside 1000) is indicated for the treatment of iron deficiency when oral iron preparations are ineffective or cannot be used.

Intravenous iron preparations must always be prescribed by brand.

1. **Dosage and frequency of use**

The dose of Monofer**®** is expressed in milligrams (mg) of elemental iron. The iron need and the administration schedule for Monofer**®** must be individually established for each patient. After an iron deficient state has been corrected, patients may require repeated therapy to maintain acceptable haemoglobin and/or iron parameters.

**For patients ≥50kg,** **dose is 20mg/kg rounded down to the nearest 100mg in the anaemic patient, with a maximum of 2000mg.** For obese patients use ideal body weight.

For dosing for patients with weight <50kg: follow the Ganzoni formula:

Iron need (mg of iron)= body weight (kg) x (150-Hb)/10 x 2.4 + 500

1. **Patient monitoring before the infusion**

- The Hb level should be checked and recorded before the first dose is given.

- A set of observations (including blood pressure, pulse rate and temperature) and the MEWS score should be measured and recorded before administration. If the patient has any signs or symptoms of infection alert the appropriate doctor. They must decide whether or not is appropriate to delay giving the dose until any infection has resolved.

1. **Method of administration**

Ensure adrenaline (epinephrine) is available prior to administration in case of an anaphylactoid reaction. Hydrocortisone 100mg IV and chlorpheniramine 10mg IV should also be prescribed as prn.

Monofer**®** is available as iron (lll) isomaltoside solution for injection containing 100mg iron in 1ml. This is available as 1ml, 5ml and 10ml ampoules.

Doses should be diluted in 100mL **sodium chloride 0.9%** (although volumes up to 500mL are acceptable where appropriate) and administered by intravenous infusion. Only sodium chloride 0.9% should be used for dilution and flushing.

Doses up to 1000mg must be administered over more than 15 minutes. Doses exceeding 1000mg must be administered over 30 minutes or more.

No other therapeutic agents should be added.

Storage and Handling:

Inspect vials visually for sediment and damage before use and only use if sediment-free.

Store vials at room temperature.

Each vial is intended for single use only. Any unused product or waste material should be discarded.

1. **Cautions, contraindications and side effects**

Parenterally administered iron preparations can cause hypersensitivity reactions including anaphylactoid reactions, which may be potentially fatal. If allergic reactions or signs of intolerance occur during administration, the treatment must be stopped immediately. Hypersensitivity reactions have also been reported after previously uneventful doses of parenteral iron complexes. The patient should be observed for adverse effects for at least 30 minutes following each Monofer**®** injection.

**FISHBANE reaction** – typically when this occurs, a patient will get a small amount of iron and will complain of chest and back pain. It is nothing more than a mild, self-limited innocent arthralgia myalgia reaction that goes away. It is never associated with hypotension, tachycardia, periorbital oedema or stridor. The management of the Fishbane reaction is to stop the infusion and the symptoms will resolve. After a period of several minutes, re-challenge the patient. It will not recur and it should not be considered a serious adverse event.

Any side effects, however minor, should be reported to the doctor. The patient should be advised to telephone the UHS medicines helpline (02381 206907) or the unit/ ward if they experience any side effects at home after the infusion.

Contraindications:

- Known hypersensitivity to the active substance, to Monofer**®** or to any of its excipients

- Known serious hypersensitivity to other parenteral iron products

- Non-iron deficiency anaemia

- Iron overload or disturbances in utilisation of iron

- Acute infection, ongoing bacteraemia or flare of a rheumatological inflammatory condition

- Decompensated liver cirrhosis and hepatitis

- Concomitant administration with oral iron preparations (allow a minimum five day gap as absorption of oral iron might be decreased)

Cautions:

- Chronic infection

- Asthma, eczema or atopic allergies

- Hypotensive episodes may occur if the intravenous injection is administered too rapidly.

- Pregnancy/lactation (seek specialist advice)

Please refer to the summary of product characteristics for a full list of contraindications, cautions and side effects.

In the event of a drug extravasation then standard procedures should be instituted (stop the infusion, aspirate residual medicines through the cannula and elevate the limb) and the Medicines Advice Service (extension 6908 and 6909) should be contacted for further advice. While the risk of tissue damage is low there is a risk of permanent skin staining.

1. **Documentation on the managed care unit**

- Record in medical notes: Attendance, clinical observations, patient status and supply of a patient information leaflet.

- Ensure HMR is written.

- Every patient who receives Monofer**®** should be provided with a patient information leaflet and details of who to contact if in the event of a possible adverse reaction.

- Once iron repletion has occurred it is the responsibility of the referring doctor to ensure regular assessments are completed to ensure that iron levels are corrected and maintained.

**Obtain required patient details:**

haemoglobin, weight and height

**Does the patient’s weight give them a BMI greater than or equal to 30?**

**Yes:** use ideal body weight for dose and maximum single dose calculations

**No:** use actual weight

**Calculate dose of iron required for full repletion:**

| **Hb (g/L)** | **Weight 35kg to <50kg** | **Weight 50kg to <70kg** | **Weight ≥70kg** |
| --- | --- | --- | --- |
| **≥100** | 500mg | 1000mg | 1500mg |
| **<100** | 1000mg | 1500mg | 2000mg |

**Is this dose more than the maximum single dose of 20mg/kg?**

**Yes:** the dose must be split over 2 infusions:

1. The 20mg/kg dose (round down to the nearest 100mg)

2. The remainder of the calculated dose given at least one week later

**No:** the full dose can be given as a single infusion

| **Height**  **(imperial)** | **Height**  **(metric)** | **Weight for BMI**  **of 30 (kg)** | **IBW (M)**  **(kg)** | **IBW (F)**  **(kg)** |
| --- | --- | --- | --- | --- |
| 5ft | 152 | 69.7 | 50 | 45.5 |
| 5ft 1in | 155 | 72.0 | 52.3 | 47.8 |
| 5ft 2in | 157 | 74.4 | 54.6 | 50.1 |
| 5ft 3in | 160 | 76.8 | 56.9 | 52.4 |
| 5ft 4in | 163 | 79.3 | 59.2 | 54.7 |
| 5ft 5in | 165 | 81.8 | 61.5 | 57 |
| 5ft 6in | 168 | 84.3 | 63.8 | 59.3 |
| 5ft 7in | 170 | 86.9 | 66.1 | 61.6 |
| 5ft 8in | 173 | 89.5 | 68.4 | 63.9 |
| 5ft 9in | 175 | 92.1 | 70.7 | 66.2 |
| 5ft 10in | 178 | 94.8 | 73 | 68.5 |
| 5ft 11in | 180 | 97.6 | 75.3 | 70.8 |
| 6ft | 183 | 100.3 | 77.6 | 73.1 |
| 6ft 1in | 185 | 103.1 | 79.9 | 75.4 |
| 6ft 2in | 188 | 106.0 | 82.2 | 77.7 |

**Appendix C – Grading and management of acute hypersensitivity reactions to intravenous iron infusions**


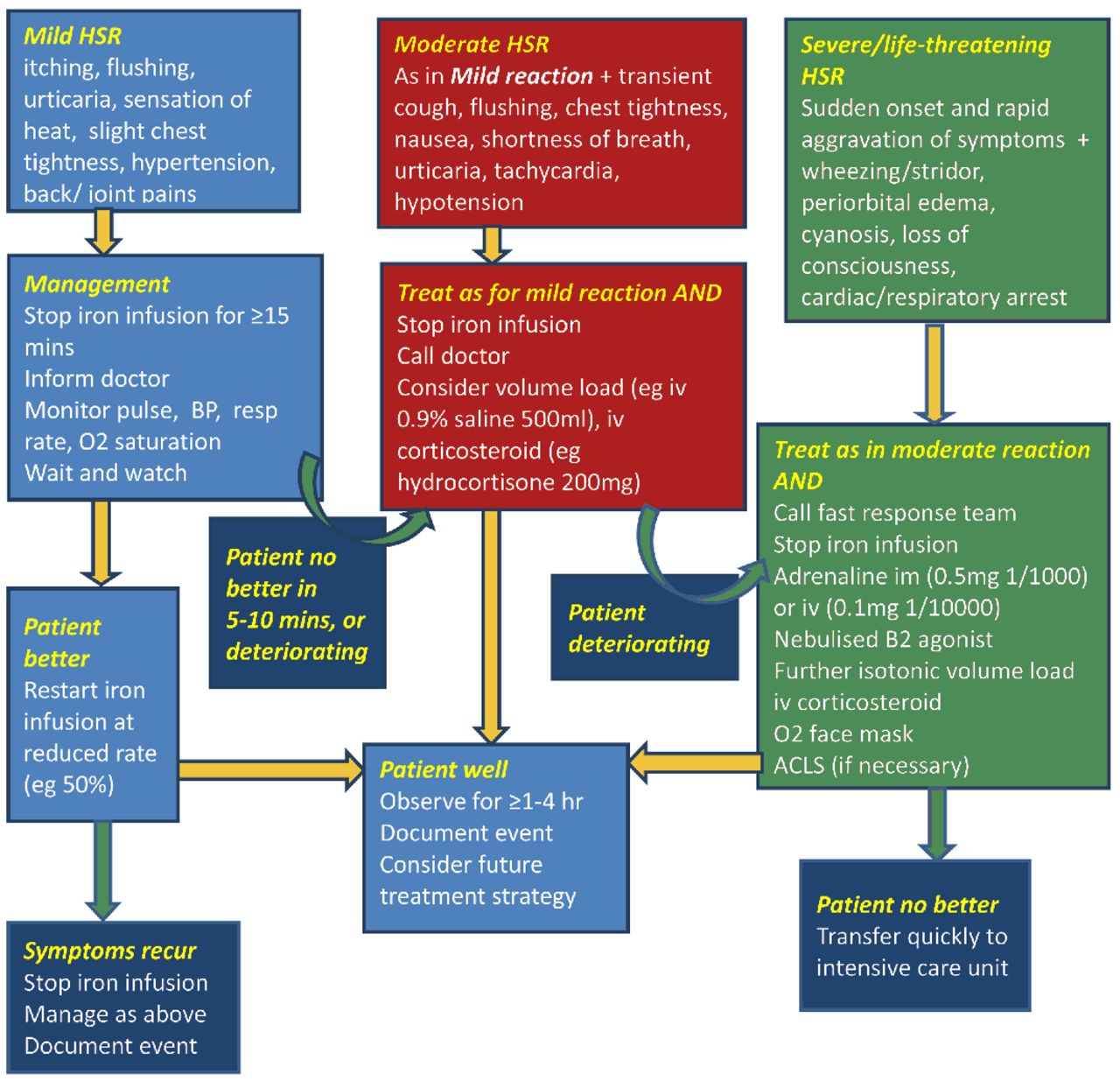


Taken from Rampton, D. et al. (2014) Hypersensitivity reactions to intravenous iron: guidance for risk minimization and management. *Haematologica* 99(11) pp. 1671-6.

<https://hosted.bmj.com/media/images/UK-MONF-0818-00017-InternationalConsensusSummaryLeavepiece-Digital-UK-Aug2018.pdf>

<https://www.ncbi.nlm.nih.gov/pubmed/27022297>

| Adult Monofer® infusion guideline for patients undergoing surgery | Version: | 1 |
| --- | --- | --- |

| Document Monitoring Information | |
| --- | --- |
| Approval Committee: |  |
| Date of Approval: |  |
| Ratification Committee: |  |
| Date of Ratification: |  |
| Signature of ratifying Committee Group/Chair: |  |
| Lead Name and Job Title of originator/author or responsible committee/individual: | Authors: Mai Wakatsuki (Consultant Anaesthetist and lead clinican for Perioperative Optimisation of Anaemia Service) |
| Policy Monitoring (Section 6) Completion and Presentation to Approval Committee: | PRG |
| Target audience: | Prescribers creating prescriptions for Monofer**®** for perioperative anaemia service Nurses administering Monofer**®**. |
| Key words: | Intravenous iron, Monofer**®**, iron(III) isomaltoside |
| Main areas affected: | The majority of Perioperative Monofer administration is expected to occur in the Surgical Day unit, Lymington Hospital, F level theatre recovery and surgical wards |
| Summary of most recent changes if applicable: | Dosing change |
| Consultation: |  |
| Equality Impact Assessment completion date: |  |
| Number of pages: |  |
| Type of document: | Guideline (level 1) |
| Does this document replace or revise an existing document | Yes |
| Should this document be made available on the public website? | No |
| Is this document to be published in any other format? | No |

The Trust strives to ensure equality of opportunity for all, both as a major employer and as a provider of health care. This document has therefore been equality impact assessed to ensure fairness and consistency for all those covered by it, regardless of their individual differences, and the results are available on request.

**Appendix 4- POAS bloods sent**

[Hb], WBC, Plts, Neutrophils, MCV, RDW %, Platelet crit (PCT), Monocytes, MCHC, Triglyceride, TSAT %, Transferrin, Iron, Creatinine, Total protein, Albumin, Thyroxine, LDL, Cholesterol, Calcium/adjusted, Ferritin, Folate, B12
